# Functional Genomic Analysis of *Aspergillus flavus* Interacting with Resistant and Susceptible Peanut

**DOI:** 10.3390/toxins8020046

**Published:** 2016-02-15

**Authors:** Houmiao Wang, Yong Lei, Liying Yan, Liyun Wan, Xiaoping Ren, Silong Chen, Xiaofeng Dai, Wei Guo, Huifang Jiang, Boshou Liao

**Affiliations:** 1 Key Laboratory of Oil Crop Biology of the Ministry of Agriculture, Oil Crops Research Institute of Chinese Academy of Agricultural Sciences, Wuhan 430062, China; wanghoumiao@163.com (H.W.); leiyong@caas.cn (Y.L.); yanliying2002@126.com (L.Y.); wanliyun@caas.cn (L.W.); renxp1972@hotmail.com (X.R.); chsl99@163.com (S.C.); peanutlab@oilcrops.cn (H.J.); 2 Chinese Academy of Agricultural Sciences-International Crop Research Institute for the Semi-Arid Tropics Joint Laboratory for Groundnut Aflatoxin Management, Oil Crops Research Institute of Chinese Academy of Agricultural Sciences, Wuhan 430062, China; 3 Institute of Agro-Products Processing Science and Technology, Chinese Academy of Agricultural Sciences, Beijing 100193, China; daixiaofeng@caas.cn (X.D.); iewguo@126.com (W.G.)

**Keywords:** *Aspergillusflavus*, peanut, interaction, aflatoxin biosynthesis, RNA-seq

## Abstract

In the *Aspergillus* *flavus (A. flavus*)–peanut pathosystem, development and metabolism of the fungus directly influence aflatoxin contamination. To comprehensively understand the molecular mechanism of *A. flavus* interaction with peanut, RNA-seq was used for global transcriptome profiling of *A. flavus* during interaction with resistant and susceptible peanut genotypes. In total, 67.46 Gb of high-quality bases were generated for *A. flavus*-resistant (af_R) and -susceptible peanut (af_S) at one (T1), three (T2) and seven (T3) days post-inoculation. The uniquely mapped reads to *A. flavus* reference genome in the libraries of af_R and af_S at T2 and T3 were subjected to further analysis, with more than 72% of all obtained genes expressed in the eight libraries. Comparison of expression levels both af_R *vs.* af_S and T2 *vs.* T3 uncovered 1926 differentially expressed genes (DEGs). DEGs associated with mycelial growth, conidial development and aflatoxin biosynthesis were up-regulated in af_S compared with af_R, implying that *A. flavus* mycelia more easily penetrate and produce much more aflatoxin in susceptible than in resistant peanut. Our results serve as a foundation for understanding the molecular mechanisms of aflatoxin production differences between *A. flavus*-R and -S peanut, and offer new clues to manage aflatoxin contamination in crops.

## 1. Introduction

*Aspergillus* *flavus* (*A. flavus*) is a globally distributed filamentous, saprophytic fungus that frequently infects oil-rich seeds of various crop species during pre- and post-harvest, with subsequent production of mycotoxins such as cyclopiazonic acid, aflatrem, and the well-known aflatoxin [1,2]. Aflatoxins are extremely carcinogenic, mutagenic, teratogenic mycotoxins associated with both acute and chronic toxicity in humans and animals [3,4]. These deleterious impacts on health are most prominent in developing countries lacking technologies to monitor and reduce aflatoxin levels in crops, and where poor storage conditions often result in greater aflatoxins accumulation in the food supply. Besides the health implications in humans and animals, *A. flavus* colonization in crops causes significant economic losses because of destroyed/reduced utilization and lower price of aflatoxin-contaminated grains [5,6].

Peanut (*Arachis* *hypogaea* L.) is a major crop vulnerable to *A.* *flavus* infection and subsequent aflatoxin contamination [7]. A number of research activities have been carried out with an emphasis on improving host resistance and various management strategies to prevent and control aflatoxin contamination in peanut [7]. Numerous genes, proteins and other regulators associated with peanut resistance to aflatoxin contamination have been identified in previous research [8,9,10,11,12,13,14,15,16,17,18,19]. Aflatoxin contamination in peanut is a systemic interaction of host plant and *A. flavus*. Morphological development and secondary metabolism capabilities of *A. flavus*, the other organism of the interactive pathosystem, directly affect aflatoxin accumulation in peanut. The management of mycotoxin contamination in crops was directly influenced by the research on the aflatoxin biosynthetic pathway. To develop effective means of combating aflatoxin contamination, it is also vital importance to research on the molecular mechanisms of the development and metabolism of *A.* *flavus* that is colonized in the peanut seed.

RNA-seqing is a rapid and high-throughput technology for transcriptomic profiling analysis, which has been used to survey sequence variations and complex transcriptomes with low false-positive rates and high sensitivity and reproducibility [20,21]. Application of RNA-seq has greatly accelerated the understanding of the complexity of gene expression, regulation, and networks of organism under various spatial-temporal conditions, and gene expression can be more accurately quantified using RNA-seq approaches than by conventional transcriptomic analysis [22]. Over the past decade, progresses on the numerous fungi have been studied intensely using RNA-seq [5,6,20,23,24,25,26,27]. For an organism with a well-annotated genome, mapping read sequences to the corresponding reference genome is the first and essential step for RNA-seq data analysis [23]. The whole-genome sequencing of *A. flavus* was completed [27], and annotation of the genome of the fungus showed various genes that are potentially related with conidial development and aflatoxin biosynthesis [28]. In addition, RNA-seq technology has been used in transcriptomic analyses of aflatoxin biosynthesis and mycelial development in *A. flavus* response to resveratrol [5], 5-azacytidine [23,29], menadione [30], water activity [31] and temperature [32].

To comprehensively understand the molecular mechanism of *A. flavus* interaction with the peanut, an RNA-seq approach was applied in this study to obtain and compare transcriptomic profiles of *A. flavus* which colonized in the resistant and the susceptible peanut seed at the whole-genome level. The dynamic differences of *A. flavus* transcriptome profiles from *A. flavus*-resistant and -susceptible peanut pathosystems were identified. The possible roles of differentially expressed genes and metabolic pathways were discussed, and the mechanism of *A. flavus* interaction with the resistant and the susceptible peanut was also deduced. In addition, the significant transcriptomic information will be helpful for further annotation of the genome of *A. flavus*. This study will also aid further understanding of aflatoxin contamination and contribute to the design of new strategies to manage aflatoxin contamination in the peanut and other crops.

## 2. Results

### 2.1. RNA-seq and Transcriptome Profiles of the A. flavus

To explore differences in *A. flavus* transcriptomes between *A. flavus*-resistant and -susceptible peanut pathosystems, resistant peanut cultivar Zhonghua 6 (R) and susceptible cultivar Zhonghua 12 (S) were selected for analysis as host plant of *A. flavus*. On the basis of difference in aflatoxin production between *A. flavus*-R peanut (af_R) and *A. flavus*-S (af_S) pathosystems, the first (T1), third (T3) and seventh (T3) days after inoculation were identified as crucial inflection time points to provide insight into genetic expression of *A. flavus* interacting with different peanut genotypes. Six samples, *i.e.*, af_R_T1, af_R_T2, af_R_T3, af_S_T1, af_S_T2 and af_S_T3, with two biological replicates, were therefore used for transcriptome sequencing and transcriptomic analysis. An overview of the sequencing is outlined in Table 1 and Table S1. After quality control, 4.80 to 6.36 Gb clean bases were obtained from each of the 12 *A. flavus*-peanut libraries, with a total of 34.54 and 32.90 Gb clean bases for af_R and af_S, respectively (Table 1). Between 2115 and 33,780,750 of these reads were uniquely mapped to the *A. flavus* reference genome, resulting in a total of 99,599,838 uniquely mapped reads for all further analysis (Table S1). The genic distribution of uniquely mapped reads indicated that most reads (>85.1%) were mapped to exons, and the others were distributed between introns (10.8%–14.3%) and intergenic regions (0.6%–0.8%) (Table S1).

**Table 1:** Summary of RNA-seq reads generated in the study.

Sample Name	Raw Reads	Clean Reads	Clean Bases (Gb)	Error Rate (%)	Q20 (%)	Q30 (%)	GC Content (%)	rRNA (%)
af1_R_T1	57,599,682	55,753,432	5.58	0.03	97.25	91.78	45.18	2.07
af2_R_T1	54,642,766	52,827,190	5.28	0.06	97.32	91.95	44.87	1.97
af1_R_T2	65,478,414	63,861,410	6.38	0.06	97.65	92.74	44.57	2.43
af2_R_T2	58,356,648	55,965,182	5.60	0.07	96.51	90.11	45.17	1.80
af1_R_T3	67,147,990	63,523,784	6.36	0.09	95.81	88.30	50.08	3.80
af2_R_T3	56,259,592	53,486,954	5.34	0.10	95.77	87.83	50.04	3.20
af1_S_T1	59,822,028	58,024,886	5.80	0.06	97.27	91.85	44.23	1.77
af2_S_T1	61,482,172	59,691,186	5.96	0.06	97.29	91.89	44.34	2.33
af1_S_T2	55,081,582	52,844,526	5.28	0.08	96.26	89.17	45.10	1.57
af2_S_T2	58,420,538	56,092,690	5.60	0.08	96.21	89.01	45.49	1.87
af1_S_T3	56,907,234	54,518,226	5.46	0.08	96.20	89.00	45.75	2.47
af2_S_T3	50,079,030	48,035,682	4.80	0.06	97.20	91.23	45.53	3.90

All mapped reads from the 12 *A. flavus* libraries were merged and assembled by Cufflinks [33]. The structure of previously annotated genes was optimized and novel genes were characterized using Cuffcompare. Structures of 51.81% (7188) of the 13,875 genes in the *A. flavus* genome database [34] were optimized and 582 novel genes were obtained (Table S2). All novel genes were compared against the National Center for Biotechnology Information (NCBI) non-redundant (Nr) protein database [35] using Blastx, 306 (52.58%) genes were searched for the corresponding homologies in Nr database (Table S2). Additionally, all 582 novel genes in this study were subjected to Gene Ontology (GO) classification, with 199 novel genes having Blast2GO (E-value = 1.0 × 10^−6^) matches to known proteins thereby assigned to a broad range of GO terms (Figure S1).

We obtained 14,457 genes, including 13,875 previously annotated ones and 582 novel ones. Using the uniquely mapped reads, the abundance of all obtained genes was normalized and calculated by the reads per kilobase per million mapped reads (RPKM) method (Table S3) [36]. Because few mycelia of *A. flavus* had colonized the pathosystems after one-day incubation, the fungal transcriptomic data from this time point represented only a small proportion of the total clean reads. The amount of reads uniquely mapped to *A. flavus* reference genome from these four libraries (af1_R_T1, af2_R_T1, af1_S_T1 and af1_S_T1) was so few (Tables S1 and S3) that they could not be used for further analysis. Distributions of gene expression levels were similar among the remaining eight *A. flavus* libraries from the *A. flavus*-R and -S pathosystems at the later two time points (3 and 7 d after incubation) (Table S4 and Figure S2). In each of the eight libraries at these two time points, more than 72% of all obtained genes (14,457) were expressed (RPKM > 1) and over 2505 genes were highly expressed (RPKM > 60) (Table S4); furthermore, the gene expression data were highly reproducible between two biological replicates in af_R and af_S at each respective time point (Table S4 and Figure S3).

### 2.2. Identification of Differentially Expressed Genes

**Figure 1:**
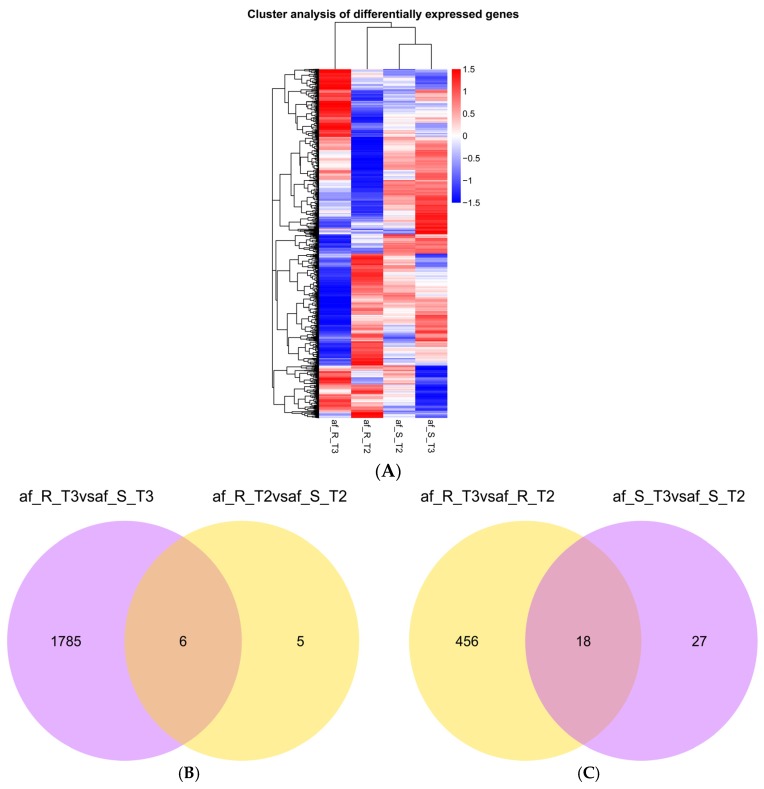
Hierarchical clustering dendrogram and Venn diagram of differentially expressed genes (DEGs) in the *A. flavus*-peanut pathosystem. (**A**) Hierarchical clustering dendrogram of DEGs obtained using the RNA-seq data from four *A. flavus* samples incubated for three and seven days based on log_10_(RPKM+1). Red and blue bands indicate high and low gene expression levels, respectively. (**B**) Venn diagram showing overlaps between DEGs at the second and the third time points in the comparisons af_R_T2 *vs.* af_S_T2 and af_R_T3 *vs.* af_S_T3. (**C**) Venn diagram of DEGs in af_R (af_R_T3 *vs.* af_R_T2) and in af_S (af_S_T3 *vs.* af_S_T2).

Differentially expressed genes (DEGs) in comparisons of af_R *vs.* af_S and T3 *vs.* T2 were identified using the DESeq R package (1.10.1, European Molecular Biology Laboratory, Heidelberg, Germany, 2010). Only those genes with the corrected *p* (*q*) value < 0.05 were considered to be differentially expressed [37]. The 1796 DEGs were detected between af_R and af_S provided clues related to the molecular mechanisms underlying *A. flavus* interaction with R and S peanut (Table 2 and Table S5), while the 501 DEGs in the comparison of T3 *vs.* T2 offered insights into the metabolic and regulatory processes in *A. flavus* during its interaction with the peanut (Table 2 and Table S5). To observe a global view of gene expression patterns, we performed hierarchical clustering of all DEGs based on log_10_(RPKM+1) for the four *A. flavus* samples of af_R and af_S incubated for three and seven days (Figure 1A). Similar expression patterns were found in two af_S samples (af_S_T2 and af_S_T3), whereas distinct sample-specific expression patterns were observed in the af_R samples (af_R_T2 and af_R_T3).

**Table 2:** Comparative analysis of differentially expressed genes (DEGs).

Comparison	Number of DEGs		
	Up-Regulated	Down-Regulated	Total
af_R_T2 *vs.* af_S_T2	9	2	11
af_R_T3 *vs.* af_S_T3	647	1144	1791
af_R_T3 *vs.* af_R_T2	317	157	474
af_S_T3 *vs.* af_S_T2	34	11	45
total	1926		

Eleven DEGs were found between af_R and af_S at T2 (af_R_T2 *vs.* af_S_T2), while 1791 DEGs were found between af_R and af_S at T3 (af_R_T3 *vs.* af_S_T3) (Table 2 and Figure 1B). Furthermore, six DEGs involved in primary metabolism of *A. flavus* exhibited common differential expression patterns in comparisons of af_R_T2 *vs.* af_S_T2 and af_R_T3 *vs.* af_S_T3 (Figure 1B). The above analysis results implied that many more DEGs were involved in metabolic and regulatory pathways of af_R and af_S at the third time point than at the second one. As shown in Figure 1C, 474 DEGs were found between T3 and T2 in af*_*R (af_R_T3 *vs.* af_R_T2) and 45 DEGs were obtained between af_S at T3 and at T2 (af_S_T3 *vs.* af_S_T2), suggesting a markedly higher number of gene expression changes in af_R than in af_S. Eighteen DEGs exhibited common differential expression patterns in comparisons of af_R_T3 *vs.* af_R_T2 and af_S_T3 *vs.* af_S_T2 (Figure 1C), implying that most DEGs in the comparison of af_R_T3 *vs.* af_R_T2 were different from those in af_S_T3 *vs.* af_S_T2.

### 2.3. Gene Ontology and Kyoto Encyclopedia of Genes and Genomes Enrichment Analysis of DEGs

To analyze the functions of DEGs, a Gene Ontology (GO) enrichment analysis was performed using the GOseq method in Blast2GO [38]. We firstly performed a GO analysis of genes differentially expressed in af_R compared with af_S at paired time points (af_R_T2 *vs.* af_S_T2 and af_R_T3 *vs.* af_S_T3, respectively). The GO functional enrichment analysis of the 1113 (62.14%) DEGs with GO annotation in the af_R_T3 *vs.* af_S_T3 revealed significantly enriched terms in the biological process and the molecular function categories (Table S6). Catalytic activity (GO: 0003824) and oxidoreductase activity (GO: 0016491) with 835 and 306 genes, respectively, were the first two dominant terms in the molecular function category, and metabolic process (GO: 0008152) with 692 genes was dominant in the biological process category. Except for nine GO terms (GO: 0022857, GO: 0022892, GO: 0015075, GO: 0015849, GO: 0046942, GO: 0005342, GO: 0046943, GO: 0055085 and GO: 0004499), all other GO terms were much more enriched in down-regulated DEGs than up-regulated ones. In contrast to the T3 comparison, the GO analysis failed to confirm enrichment in any term using the differentially expressed data obtained from the comparison of af_R_T2 with af_S_T2. The GO analysis revealed that a larger number of repressed responses were obtained in af_R compared with af_S at T3. Additionally, We performed GO enrichment analysis of DEGs between T3 and T2 in af_R and af_S (af_R_T3 *vs.* af_R_T2 and af_S_T3 *vs.* af_S_T2), respectively. Of the 263 (55.48%) DEGs with GO annotation in af_R_T3 *vs.* af_R_T2, only two GO terms (GO: 0003824 and GO: 0016491) in the molecular function category and three (GO: 0044710, GO: 0055114 and GO: 0005975) in the biological process category were significantly enriched (Table S5). Other than the carbohydrate metabolic process (GO: 0005975), substantially more up-regulated DEGs than down-regulated ones were enriched in the other four terms. But the analysis failed to identify any term enriched in DEGs between af_S_T3 and af_S_T2. The dynamic changes in gene expression were observed between T3 and T2 in af_R and af_S, respectively. The GO analysis results suggested that a greater number of active responses take place between different interactive times in af_R than in af_S.

**Figure 2:**
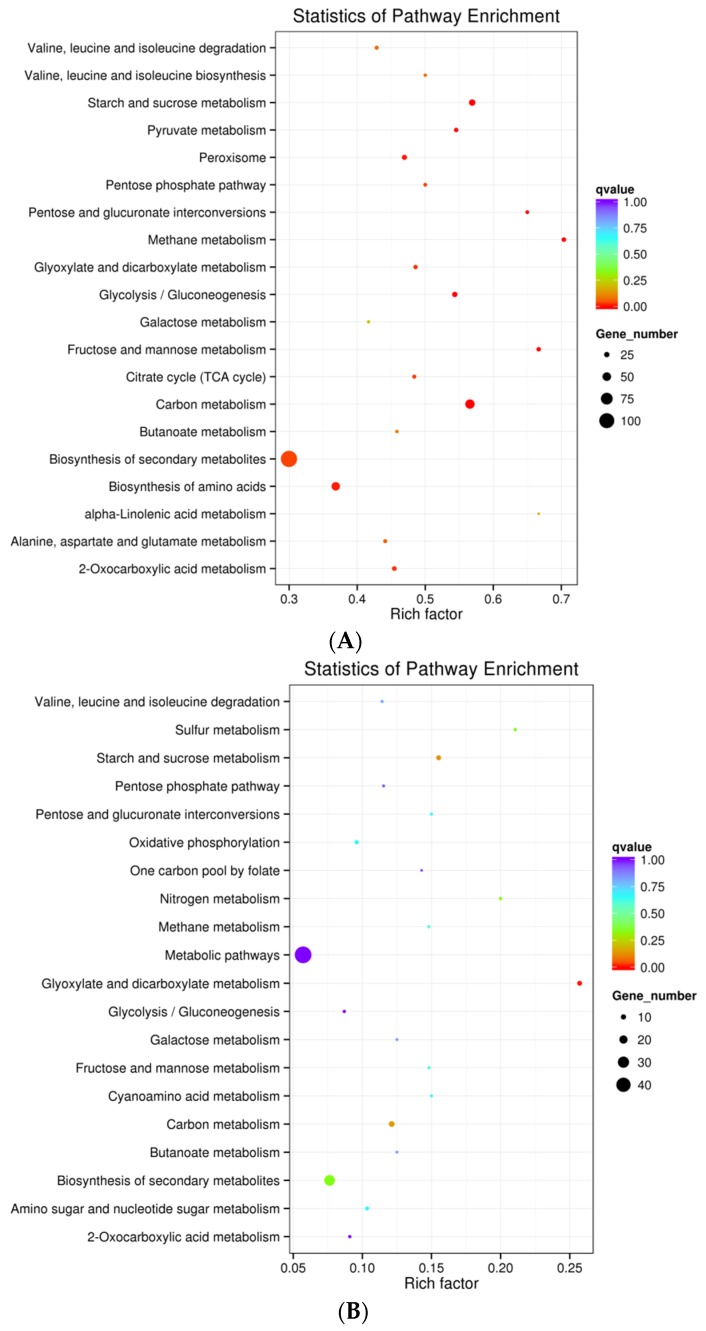
Scatterplot of Kyoto Encyclopedia of Genes and Genomes (KEGG) pathways enriched in DEGs between different *A. flavus* samples: results of KEGG analysis of comparisons (**A**) af_R_T3 *vs.* af_S_T3 and (**B**) af_R_T3 *vs.* af_R_T2. The rich factor is the ratio of the number of DEGs to the total number of genes in a given pathway. Dot sizes and colors correspond to the number of genes and the range of the corrected *p* (*q*) value, respectively.

To further investigate the biological functions and interactions of genes, a Kyoto Encyclopedia of Genes and Genomes (KEGG) pathway enrichment analysis was conducted using KEGG Orthology Based Annotation System (KOBAS) (v2.0, Center for Bioinformatics, Peking University, Beijing, China, 2014) [39]. All DEGs obtained from comparisons af_R_T2 *vs.* af_S_T2, af_R_T3 *vs.* af_S_T3, af_R_T3 *vs.* af_R_T2 and af_S_T3 *vs.* af_S_T2 were analyzed to identify their associated KEGG metabolic pathways. Consistent with the results of the GO analysis, no KEGG pathways were significantly enriched (*q* value < 0.05) in DEGs data obtained from af_R_T2 *vs.* af_S_T2 and af_S_T3 *vs.* af_S_T2. We found that 14 pathways were significantly enriched in DEGs between af_R_T3 and af_S_T3, including 12 pathways involved in the supply of nutrient and energy for fungal development, biosynthesis of secondary metabolites (afv01110) and peroxisomes (afv04146) (Figure 2A). Twenty-three genes were differentially expressed in peroxisome (afv04146), providing evidence that various oxidative reactions were also differentially regulated between af_R and af_S. Additionally, only the glyoxylate and dicarboxylate metabolism pathway (afv00630) was significantly enriched for DEGs between af_R_T3 and af_R_T2 (Figure 2B). Although several DEGs involved in secondary metabolites biosynthesis were identified in the comparison of af_R_T3 *vs.* af_R_T2, these DEGs were not enriched in the biosynthesis of secondary metabolites pathway (afv01110).

### 2.4. Expression Analysis of Development-Related Genes and Secondary Metabolism Gene Clusters in A. flavus

By analyzing gene expression pattern data obtained from deep sequencing, especially those in the list of the 1926 genes that were significantly differentially transcribed (Table S5), we found that the expression of some genes involved in development and secondary metabolism of *A. flavus* was significantly changed when the fungus interacted with R and S peanut (Table 3 and Table S7). Seventeen DEGs involved in degradation of plant cell walls were observed in the experiment (Table 3). All 17 of these DEGs were significantly down-regulated in af_R_T3 *vs.* af_S_T3, while only one DEG (AFLA_062930) encoding the alpha-N-arabinofuranosidase was identified in af_S_T3 *vs.* af_S_T2 and it was up-regulated in this comparison. However, no mycelial growth-related DEGs were observed in neither af_R_T2 *vs.* af_S_T2 nor af_R_T3 *vs.* af_R_T2. Concurrently, transcriptions of conidia-specific genes, such as conidial hydrophobin RodA/RolA (AFLA_098380), conidiation-specific proteins (AFLA_083110, AFLA_112100, AFLA_04479, and AFLA_044800) and conidial development related genes such as AtfA (AFLA_031340) and PksP (AFLA_00617), were significantly changed to various degrees in different comparisons (Table 3). Six of these DEGs (AFLA_098380, AFLA_083110, AFLA_112100, AFLA_04479, AFLA_031340 and AFLA_044800) were significantly down-regulated in af_R_T3 *vs.* af_S_T3 and five (AFLA_098380, AFLA_083110, AFLA_04479, AFLA_044800 and AFLA_00617) were up-regulated in af_R_T3 *vs.* af_R_T2. However, no DEGs involved in conidial development were found in both af_R_T2 *vs.* af_S_T2 and af_S_T3 *vs.* af_S_T2.

**Table 3:** Differentially expressed genes related to the mycelial development and aflatoxin biosynthesis in *A. flavus.*

Gene Name	Log_2_ (fold change)				Description
	C1	C2	C3	C4	
**Mycelia**					
AFLA_105900	/	−2.6	/	/	feruloyl esterase C
AFLA_110270	/	−2.3	/	/	feruloyl esterase B-1
AFLA_128870	/	−1.6	/	/	feruloyl esterase B-2
AFLA_115930	/	−0.8	/	/	terrelysin
AFLA_023340	/	−2.3	/	/	pectinesterase A
AFLA_020000	/	−3.0	/	/	pectinesterase
AFLA_104300	/	−1.1	/	/	alpha-N-arabinofuranosidase B
AFLA_062930	/	−1.8	/	1.6	alpha-N-arabinofuranosidase 2
AFLA_063490	/	−2.1	/	/	alpha-L-arabinofuranosidaseaxhA
AFLA_070020	/	−1.3	/	/	alpha-N-arabinofuranosidase C
AFLA_089770	/	−3.8	/	/	alpha-N-arabinofuranosidase A
AFLA_038730	/	−1.7	/	/	mannan endo-1,4-beta-mannosidase E
AFLA_128610	/	−1.2	/	/	beta-mannosidase A
AFLA_117830	/	−0.7	/	/	beta-mannosidase B
AFLA_086360	/	−2.8	/	/	exopolygalacturonase C
AFLA_131770	/	−1.2	/	/	exopolygalacturonase X
AFLA_096690	/	−3.3	/	/	galacturan 1,4-alpha-galacturonidase A
**Conidia**					
AFLA_083110	/	−2.0	4.2	/	conidiation-specific protein Con-10
AFLA_112100	/	−0.4	/	/	conidiation-specific protein Con-8
AFLA_044790	/	−3.3	3.7	/	conidiation-specific family protein
AFLA_044800	/	−1.8	6.5	/	conidiation protein Con-6
AFLA_098380	/	−0.6	1.7	/	conidial hydrophobinRodA/RolA
AFLA_031340	/	−0.6	/	/	transcription factor AtfA
AFLA_006170	/	/	2.2	/	polyketidesynthetasePksP
**Aflatoxin**					
AFLA_139160	/	−0.5	/	/	aflX/ordB/monooxygenase/oxidase
AFLA_139210	/	/	1.2	/	aflP/omtA/O-methyltransferase A
AFLA_139260	/	/	1.2	/	aflG/avnA/P450 monooxygenase
AFLA_139270	/	−0.6	/	/	aflNa/hypD/hypothetical protein
AFLA_139280	/	/	1.2	/	aflN/verA/monooxygenase
AFLA_139380	/	/	1.2	/	aflA/fas-2/fatty acid synthase alpha subunit
AFLA_139390	/	−1.4	/	/	aflD/nor-1/reductase
AFLA_139400	/	/	1.1	/	aflCa/hypC/hypothetical protein
AFLA_139410	/	/	1.2	/	aflC/pksA/polyketide synthase
AFLA_112820	/	−1.4	/	/	toxin biosynthesis ketoreductase, putative
AFLA_050450	/	−1.2	/	/	toxin biosynthesis protein

C1, C2, C3 and C4 refer to the comparisons af_R_T2 *vs.* af_S_T2, af_R_T3 *vs.* af_S_T3, af_R_T3 *vs.* af_R_T2 and af_S_T3 *vs.* af_S_T2, respectively. af_R: the *A. flavus*-R peanut pathosystem; af_S: *A. flavus*-S peanut pathosystem. T1, T2 and T3 indicates the first, third and seventh days after incubation of these pathosystems. The symbol “/” indicates that the gene was not differentially expressed in a given comparison.

Of 56 secondary metabolism gene clusters [40], 36 had at least one DEG in one or more comparisons, corresponding to a total of 91 DEGs including eight backbone genes (AFLA_006170, AFLA_017840, AFLA_060680, AFLA_066980, AFLA_079400, AFLA_105190, AFLA_114820 and AFLA_139410) (Table S7). One, 79, 20 and three DEGs were found in af_R_T2 *vs.* af_S_T2, af_R_T3 *vs.* af_S_T3, af_R_T3 *vs.* af_R_T2 and af_S_T3 *vs.* af_S_T2, respectively. Some of these DEGs were differentially expressed in two or three different comparisons. Additionally, the 54#, 9# and 26# cluster with eight, seven and six DEGs, respectively, were the first three dominant ones in these 36 secondary metabolism gene clusters; while 14 of the 36 clusters only possessed one DEG. The aflatoxin biosynthetic pathway cluster (54#) was most worthy focused on because the carcinogenic, mutagenic aflatoxin has been characterized in *A. flavus* [2]. The transcriptions of 32 genes in 54# cluster were down-regulated to various degrees in af_R_T2 *vs.* af_S_T2, but none were differentially expressed between these two pathosystems. In af_R_T3 *vs.* af_S_T3, 18 down- and 15 up-regulated genes in 54# cluster were obtained. Interestingly, three genes (AFLA_139160, AFLA_139270 and AFLA_139390) were significantly down-regulated. Surprisingly, we also identified other two significantly down-regulated genes (AFLA_112820 and AFLA_050450) involved in aflatoxin biosynthesis (Table 3 and Table S7). Six up-regulated DEGs (AFLA_139210, AFLA_139260, AFLA_139280, AFLA_13938, AFLA_139400 and AFLA_139410) were found in 54# cluster between af_R_T3 and af_R_T2, whereas no DEGs were found in this cluster between af_S_T3 and af_S_T2 (Table 3). Additionally, the expressions of the other three genes (AFLA_139230, AFLA_139240 and AFLA_139500) in 54# cluster were too low in all four samples (af_R_T2, af_R_T3, af_S_T2 and af_S_T3) to be distinguished by HTSeq (Table S7).

## 3. Discussion

*Aspergillus* *flavus* is a ubiquitous saprophytic fungus that can infect agricultural crops, especially oil-rich crops such as peanut both in pre- and post-harvest and bring about aflatoxin contamination [2,41]. As aflatoxins are toxic and carcinogenic mycotoxins and *A. flavus* is also an opportunistic pathogen of the human [2,42], controlling aflatoxin contamination in the peanut is vital. Previous research has focused on the host plant and has revealed many genes and molecules that control the intricate process of aflatoxin biosynthesis in *A. flavus* [2,5,7,8,10,43,44,45,46]. While aflatoxin contamination in peanut is a systemic interaction of *A. flavus* and its host, the development and metabolism of *A. flavus* also directly influence aflatoxin accumulation in peanut. The developmental and metabolic mechanism of *A. flavus* interaction with peanut were firstly discussed in this study. To obtain a broad perspective on the molecular mechanisms of *A. flavus* interaction with peanut, RNA-seq was applied to identify transcripts differentially expressed in *A. flavus* interacting with the resistant *vs.* the susceptible peanut seed. A total of 14,457 genes were obtained, including 13,875 previously annotated genes and 582 novel ones. These results enriched the genomic information on *A. flavus* in public databases, and laid a foundation for the evaluation and understanding of *A. flavus* interaction with peanut.

The *A. flavus* genes differentially expressed between *A. flavus*-R and -S peanut pathosystems included those related to mycelial and conidial development, aflatoxin biosynthesis, various enzymes, expressed and hypothetical proteins as well as proteins of unknown function. A GO analysis, which classifies genes into biological process, molecular function and cellular component categories, demonstrated a clear distinction among different comparisons. Noticeably, most GO terms were more highly enriched in down-regulated DEGs than up-regulated ones in af_R_T3 *vs.* af_S_T3, and the down-regulated gene *aflX/ordB* (AFLA_139160) of the aflatoxin biosynthetic cluster (54#) was significantly enriched in oxidoreductase activity (GO: 0016491). The *aflX/ordB* gene (AFLA_139160) encoding a monooxygenase participates in aflatoxin biosynthesis [47]. By contrast, substantially more up-regulated than down-regulated DEGs were enriched in af_R_T3 *vs.* af_R_T2. Interestingly, *aflA/fas-2* (AFLA_139380), *aflG/avnA* (AFLA_139260), *aflN/verA* (AFLA_139280) and *aflP/omtA* (AFLA_139210) of the 54# cluster, which were differentially up-regulated in af_R_T3 *vs.* af_R_T2, were enriched in different biological process (GO: 0044710, GO: 0055114 and GO: 0005975) and molecular function (GO: 0003824 and GO: 0016491) categories. Similar to the results of the GO analysis, several KEGG pathways were enriched in DEGs between af_R_T3 and af_S_T3 and between af_R_T3 and af_R_T2, whereas no KEGG pathways were significantly enriched in comparisons of af_R_T2 *vs.* af_S_T2 or af_S_T3 *vs.* af_S_T2. The biosynthesis of secondary metabolites (afv01110) was significantly enriched in DEGs between af_R_T3 and af_S_T3; moreover, this pathway was enriched in DEGs AFLA_069370, AFLA_070820 and AFLA_116080 in secondary metabolite clusters 24#, 25# and 41#, respectively. The aflatoxin biosynthetic pathway is a complex secondary metabolic process that is regulated and influenced by over 30 genes in the *A. flavus* genome [2,47,48,49]. Although several DEGs involved in the 54# cluster were found in comparisons of af_R_T3 *vs.* af_S_T3 and af_R_T3 *vs.* af_R_T2, the biosynthesis of secondary metabolites (afv01110) pathway was not found to be enriched in these DEGs. Taken together, these results implied that a greater number of repressed responses took place in af_R compared with af_S, while many more activated responses in af_R than in af_S as the interactive time increased in the *A. flavus*-peanut pathosystem.

Nutrients are indispensable elements required for the growth and metabolism of all living organisms, including plants and pathogens. For successful infection of the host plant and establishment of disease, fungal pathogens have evolved complex regulatory mechanisms to facilitate penetration, colonization and absorb nutrition for development and metabolisms, meanwhile to protect themselves against host defensive responses [50,51,52,53]. Plant cell walls, predominantly composed of cellulose/hemicellulose and pectin, are the first line of defense against bacterial and fungal pathogens [54]. Pathogens can secrete an array of polysaccharide-degrading enzymes such as feruloyl esterase [45,47], pectinesterase [55], arabinofuranosidase [51], mannosidase and galacturonidase to penetrate and degrade plant cell wall [56]. Feruloyl esterase (AFLA_105900, AFLA_110270 and AFLA_128870), pectinesterase (AFLA_020000 and AFLA_023340), arabinofuranosidase (AFLA_104300, AFLA_062930, AFLA_063490, AFLA_070020 and AFLA_089770), mannosidase (AFLA_038730, AFLA_128610 and AFLA_117830), polygalacturonase (AFLA_131770 and AFLA_086360) and galacturonidase (AFLA_096690) were significantly down-regulated in af_R compared with in af_S at the seventh day after infection. The result might imply that the S peanut seed is more conductive to invasion and colonization by *A. flavus* than the R, namely, mycelia of *A. flavus* can much more easily penetrate the S than the R peanut seed.

Aflatoxins are biosynthesized through several enzymatic reactions in mycelia of *Aspergillus* [48,49] and then the mycotoxins are exported by vesicles and vacuoles to the environment such as the host plant and the medium [57,58,59,60]. In af_R_T3 *vs.* af_S_T3, five down-regulated DEGs (AFLA_139160, AFLA_139270, AFLA_139390, AFLA_112820 and AFLA_050450) involving in aflatoxin biosynthesis were found. Among them, *aflX/ordB* (AFLA_139160), *aflNa/hypD* (AFLA_139270) and *aflD/nor-1* (AFLA_139390) belong to the aflatoxin biosynthetic cluster. The oxidoreductase Nor-1 together with NorA and NorB reduce norsolorinic acid, the first stable intermediate in the aflatoxin biosynthesis, to averantin [49]. The enzymes hypD (monooxygenase) and ordB (oxidoreductase) may act in the formation of versicolorin B, the common precursor of producing aflatoxin B1 and B2 [61]. Expression levels of these aflatoxin biosynthesis-related DEGs were lower in af_R than in af_S after incubation for seven days, thereby explaining why aflatoxin accumulation was much less in R than in S peanut. Additionally, six DEGs (*aflA*, *aflC*, *aflG*, *aflP*, *aflN* and *aflCa*) in the 54# cluster were up regulated in af_R_T3 compared with af_R_T2. The first step in aflatoxin biosynthesis is the reaction of acetyl-CoA and malonyl-CoA catalyzed by Fas-1/aflB and aflA/Fas-2 to form the starter unit hexanoate [62], followed by conversion catalyzed by aflC/PksA to produce apolyketide, norsolorinic acid anthrone [49]. Consequently, norsolorinic acid anthrone is oxidized by aflCa/HypC to form the anthraquinone norsolorinic acid, the first stable intermediate in aflatoxin biosynthesis [63]. P450 monooxygenase (*aflG/avnA*), monooxygenase (*aflN/verA*) and O-methyltransferase A (*aflP/omtA*) enzymatic reactions are respectively involved in the conversion of averantin to 5-hydroxyaverantin, versicolorin A to demethyl-sterigmatocystin and sterigmatocystin to O-methyl sterigmatocystin in aflatoxin biosynthetic pathway [47,48]. Although no DEGs related to aflatoxin biosynthesis were obtained between af_S_T3 and af_S_T2, the transcriptions of 27 genes in the 54# cluster were activated to various degrees in this comparison.

Conidia, the asexual reproductive structure of *A. flavus*, germinate as mycelia to colonize the host plant. The survival ability of *A. flavus* conidia under severe environmental conditions is stronger than that of mycelia [64]. In addition, the colonization sphere of *A. flavus* dominantly depends on the dispersal of conidia by air, water and soil movement, rain splash and biotic factors [2]. Previous experiments have shown that both R and S peanut seeds are susceptible to seed invasion by *A. flavus* [10], with the area covered by conidia on the R seed surface similar to that on the S seed. Although conidia in the seed cotyledon interiors have not been quantitatively analyzed because of restrictions of current experimental technology, *A. flavus* may form conidia in cavities or intercellular spaces of the cotyledon. The formation of conidia in *A. flavus* requires the concerted activity of numerous signaling proteins and transcription factors [29]. Transcription of the conidial hydrophobin RodA/RolA (AFLA_098380) and conidiation-specific proteins (AFLA_083110, AFLA_112100, AFLA_044790 and AFLA_044800) [47] were significantly down-regulated in af_R_T3 compared with af_S_T3. Except for these above conidia-specific genes, *atfA* (AFLA_031340) was also down-regulated in af_R_T3 *vs.* af_S_T3. Interestingly, AtfA, as a bZIP transcription factor, possesses important functions in conidial development [65] and stabilization of asexual spores against oxidative and heat stresses [66]. Considering the results of the conidia-related DEGs analysis, we speculated that conidia might be more abundant in the interior of S seed cotyledons than in those of R. Many further experiments will be applied to verify this hypothesis. By comparing in af_R_T3 *vs.* af_R_T2, 5 up-regulated DEGs related to conidial development were obtained, including 4 conidia-specific genes (AFLA_083110, AFLA_044790, AFLA_044800 and AFLA_098380) and one conidial yellow-pigment biosynthesis-related gene *pksP*/*alb1* (AFLA_006170). The *pksP*/*alb1* gene encodes a polyketide synthase (PksP) involved in the first step of conidial pigment biosynthesis. PksP catalyzes the reaction of acetyl coenzyme A (acetyl-CoA) andmalonyl-CoA to form the heptaketide naphthopyrone [67,68]. Our results suggested that the higher abundance of conidia in af_R_T3 compared with af_R_T2 might be associated with these 5e up-regulated DEGs involved in conidial development. At the same time, the sensitive regulation of these conidial development-related genes might be reflected in their contrasting expressions between T3 and T2 in af_R than in af_S. It is worth noting that those DEGs encoding proteins of unknown functions, which may play certain roles in *A. flavus* mycelial growth, conidial formation and aflatoxin production during infection and colonization the peanut need to be uncovered in order to paint a complete picture of the interactive mechanism of *A. flavus* with peanut. This comprehensive transcriptional profiling of *A. flavus* during interaction with the peanut should advance our fundamental understanding of the various associated genes and major metabolic pathways, thereby providing a direction for further study on the management of aflatoxin contamination in crops.

## 4. Conclusions

In this study, an RNA-seq approach was employed for the first time to investigate molecular events involved in the development and metabolism of *A. flavus* during the fungus interaction with the peanut. The research demonstrated that the global transcriptional analysis provided an exhaustive view of genes involved in development of mycelia and asexual spores, controlling of biosynthesis and activities of enzymes, conidial pigments and secondary metabolites processes, which were coordinately influenced in *A. flavus* by its host peanut (R and S genotypes). The transcriptome comparisons revealed that DEGs associated with mycelial growth and penetration, conidial formation and development, and aflatoxin biosynthesis and accumulation were up-regulated in af_S compared with af_R. This differential transcription may explain why aflatoxin accumulation was much higher in *A. flavus-*S peanut pathosystem than in *A. flavus-*R. However, further research is required to determine whether these DEGs are the genes responsible for the difference in aflatoxin accumulation between *A. flavus-*R and *A. flavus-*S pathosystems. Further functional exploration of these genes may provide useful information for their future application in the management of aflatoxin contamination in crops.

## 5. Experimental Section

### 5.1. Aspergillus flavus Strain and Culture Conditions

The AF2202 strain of toxigenic *A. flavus* was maintained in 20% glycerol at −80 °C at the Oil Crops Research Institute of the Chinese Academy of Agricultural Sciences (CAAS-OCRI). To prepare the *A. flavus* inoculation, the stored conidia of AF2202 were cultured on the potato dextrose agar medium for 7 d at 29 ± 1 °C. The fresh conidia were then collected and suspended in sterile water containing 0.05% Tween-80. The concentration of conidia in the suspension was determined using a hemocytometer [5].

The peanut cultivars Zhonghua 6 and Zhonghua 12 were cultivated and supplied by CAAS-OCRI (Wuhan, China). The mature seeds of both Zhonghua 6 and Zhonghua 12 are susceptible to seed invasion by *A. flavus* at post-harvest (Figure S4), while Zhonghua 6 is resistant and Zhonghua 12 is susceptible to aflatoxin production in post-harvest seed [10]. Healthy, mature, harvested seeds of Zhonghua 6 (R) and Zhonghua 12 (S) were selected for experiments. All seeds were surface sterilized by immersion in 70% ethanol for 1.0 min and rinsed three times with sterile distilled water for 5.0 min each; then 0.5 mL of spore suspension (4.0 × 10^6^ CFU/mL) was then directly added to 10.0 g of peanut seeds in a sterile Petri plate. The inoculated samples were placed in an incubator and cultured at 29 ± 1 °C in darkness. After incubation for 1, 3 and 7 d, the *A. flavus*-colonized seeds were taken out to test aflatoxin content (five replications) by high-performance liquid chromatography [10] and to extract RNA (two replications).

### 5.2. RNA Isolation and cDNA Library Construction

Although aflatoxin production trends differed between *A. flavus*-R and -S peanut pathosystems, the aflatoxin content of these pathosystems was initially tested at the 2nd day after incubation. Aflatoxin content increased at maximum rate between the 3rd and the 4th day, and then remained stable after the 7th day in both peanut cultivars (our unpublished data). Beginning on the 2nd day, the aflatoxin content of the *A. flavus*-R pathosystem was far lower than that of the *A. flavus*-S; at its peak, the aflatoxin content of *A. flavus*-S was over 10-fold that of *A. flavus*-R. These differences in aflatoxin production between the two pathosystems suggested that the genetic expression of *A. flavus* was affected by its colonized host peanut. The 1st, 3rd and 7th day as the inflection time points (Table S8) in the process of *A. flavus* interacting with peanut is very crucial; therefore, we sampled the *A. flavus*-peanut seeds when the pathosystems were incubated for 1, 3 and 7 d to isolate RNA and construct cDNA libraries for the RNA-seq analysis. Two replicates were prepared for each sample, thereby yielding 12 libraries that were used for transcriptome sequencing on the Illumina HiSeq2000 platform (HiSeq2000, Illumina, San Diego, CA, USA, 2010).

Total RNA from the *A. flavus*-peanut pathosystem was isolated using an RNeasy^®^ Plant Mini kit (Qiagen, Shanghai, China), according to the manufacturer’s protocol. All RNA samples were treated with RNase-free DNase I. A NanoDrop^®^ 2000 spectrophotometer (Thermo Scientific, Wilmington, DE, USA), a Qubit^®^Fluorometer 2.0 (Life Technologies, Carlsbad, CA, USA) and an Agilent 2100 bioanalyzer (Agilent Technologies, Santa Clara, CA, USA) were used to test the concentration and integrity of RNA samples, and confirm that all RNA samples had an integrity value > 6.5. RNA quality detection, cDNA libraries construction and RNA sequencing were performed at the Novogene Bioinformatics Technology Co. Ltd. (Beijing, China) according to previously described methods [5,23].

### 5.3. Mapping of Reads to the Reference Genome and Quantification of Gene Expression

Raw data (raw reads) in fastq format were first processed using in-house perl scripts. Clean data (clean reads) were obtained by removing low-quality reads and those containing adapters, poly-N tails from the raw data. The Q20 and Q30 values, GC content, and sequence duplication levels were calculated for the clean data. All downstream analysis used the clean data with high quality. The sequencing data generated in this study have been deposited at the NCBI Short Read Archive database and are accessible through SRA series accession number SRP065525 (BioProject ID: PRJNA300619).

The genome and gene annotation files of *A. flavus* were downloaded directly from the Ensembl Genomes website [69]. After construction of an index from the reference genome using Bowtie (v2.0.6, Center for computational biology, Johns hopkins university, Baltimore, MD, USA, 2013) [70], paired-end clean reads were aligned to the reference genome using Tophat (v2.0.7, Center for computational biology, Johns hopkins university, Baltimore, MD, USA, 2013) with “mismatch 2” as the parameter [71]. The reference annotation based transcript assembly method in the Cufflinks (v2.1.1, Cole Trapnell’s lab in Washington University, Washington University, Washington, MO, USA, 2013) was used to construct and identify known and novel transcripts from the TopHat alignment results [33]. HTSeq (v0.5.3p9url, European Molecular Biology Laboratory, Heidelberg, Germany, 2012) was used to count the read numbers mapped to each gene. The RPKM value of each gene was then calculated based on the length of the gene and the read count mapped to this gene [36].

### 5.4. Identification and Enrichment Analysis of Differentially Expressed Genes

Statistical analyses for discovering differentially expressed genes (DEGs) were performed with the DESeq R package (v1.10.1, European Molecular Biology Laboratory, Heidelberg, Germany, 2010). To evaluate the individual effects of the host peanut (Zhonghua 6 and Zhonghua 12) and time points (T1–T3), a multifactorial analysis was conducted using the multi-factor designs method of DESeq [37]. This method evaluates the weight of each factor considered in the analysis and its impact on DEGs, according to an adjusted *p* (*q*) value < 0.05.

GO enrichment analysis of DEGs was implemented using GOseq (Release 2.12, Walter and Eliza Hall Institute of Medical Research, Parkville, Australia, 2013) [38] with a correction for gene length bias included. GO terms with a corrected *p* (*q*) value < 0.05 were considered to be significantly enriched in DEGs. KOBAS software (v 2.0, Center for Bioinformatics, Peking University, Beijing, China, 2014) [39] was used to test the statistical enrichment of DEGs in KEGG pathways. The KEGG term with a corrected *p* (*q*) value < 0.05 was considered to be significantly enriched in DEGs [72].

## Supplementary Materials

The following are available online at www.mdpi.com/2072-6651/8/2/46/s1. Figure S1: Gene Ontology (GO) classification of novel genes identified in this study. The 199 annotated novel genes were classified into three GO functional categories: biological process, cellular component and molecular function. Figure S2: Boxplot of the log-transformed RPKM expression values across four *A**. flavus* samples. RPKM: reads per kilo bases per million mapped reads. The solid horizontal line represents the median and the box encompasses lower and upper quartiles. Figure S3: Results of the pearson correlation analysis of biological replicates. Figure S4: The morphological observation of *A**. flavus*-peanut pathosystems at 7 days after incubation. Table S1: Summary of RNA-sequencing data mapped to the *A**. flavus* reference genome. Table S2: Gene structure optimization and novel gene identification. Table S3: Expression levels of all expressed genes across the 12 *A**. flavus* samples. Table S4: Distribution of gene expression levels in *A**. flavus*. Table S5: Differentially expressed genes between different comparisons of *A**. flavus* samples. An adjusted *p* (*q*) value <0.05 was set as the threshold for significant differential expression. Table S6: Results of Gene Ontology (GO) enrichment analysis of differentially expressed genes (DEGs) between different comparisons of *A**. flavus* samples. GO terms with a corrected *p* (*q*) value <0.05 were considered to be significantly enriched in DEGs. Table S7: Gene expression and differentially expressed genes (DEGs) in 55 secondary metabolic gene clusters of *A**. flavus*. C1, C2, C3 and C4 refer to the comparisons af_R_T2 *vs.* af_S_T2, af_R_T3 *vs.* af_S_T3, af_R_T3 *vs.* af_R_T2 and af_S_T3 *vs.* af_S_T2, respectively. The log_2_(foldchange) of DEGs (*q* value <0.05) in each comparison are shown in the table. Table S8: The aflatoxin content in *A. flavus*-Zhonghua 6 and *A. flavus*-Zhonghua 12 pathosystems at different days after incubation.
