# Supplementary material for: Functional Genomic Analysis of Aspergillus flavus Interacting with Resistant and Susceptible Peanut
Source: Toxins (Basel). 2016 Feb 15;8(2):46. doi: 10.3390/toxins8020046 (PMC4773799; doi:10.3390/toxins8020046)
Supplement: Supplementary file 1 [file toxins-08-00046-s001.zip › toxins-114703-supplementary/toxins-114703-supplementary.pdf]

Supplementary Materials: Functional Genomic Analysis of *Aspergillus flavus* Interacting with Resistant and Susceptible Peanut

Houmiao Wang, Yong Lei, Liying Yan, Liyun Wan, Xiaoping Ren, Silong Chen, Xiaofeng Dai, Wei Guo, Huifang Jiang and Boshou Liao

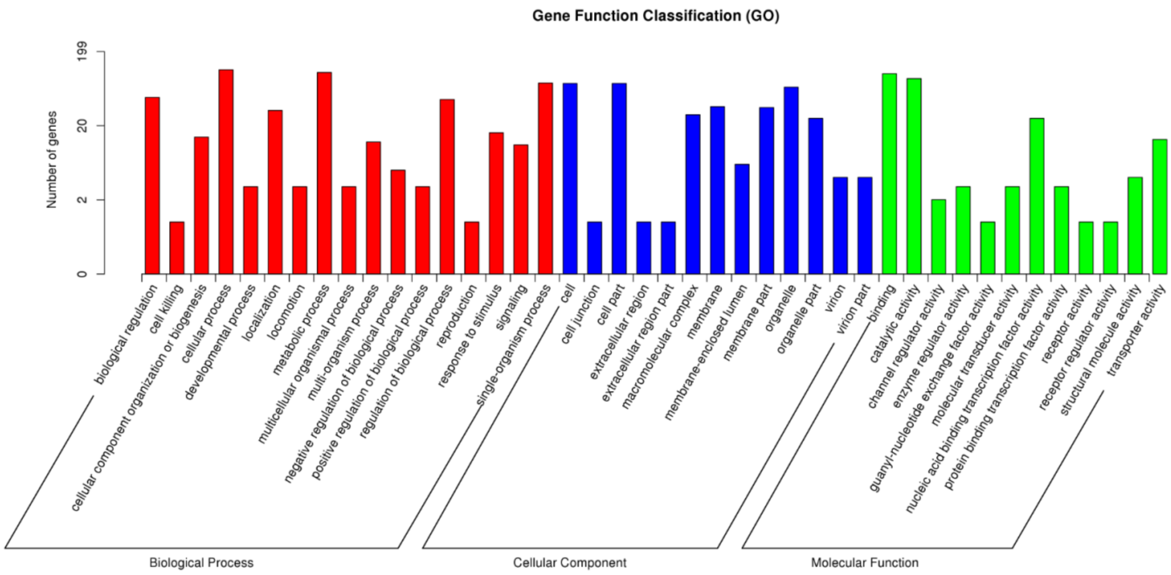

Figure S1. Gene Ontology (GO) classification of novel genes identified in this study. The 199 annotated novel genes were classified into three GO functional categories: biological process, cellular component and molecular function.

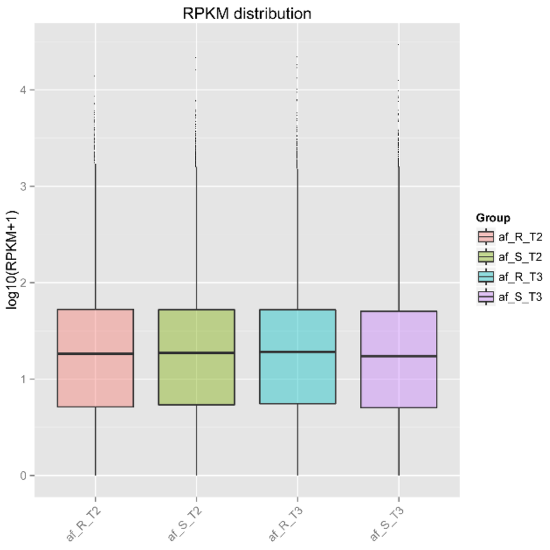

Figure S2. Boxplot of the log-transformed RPKM expression values across four *A. flavus* samples. RPKM: reads per kilo bases per million mapped reads. The solid horizontal line represents the median and the box encompasses lower and upper quartiles.

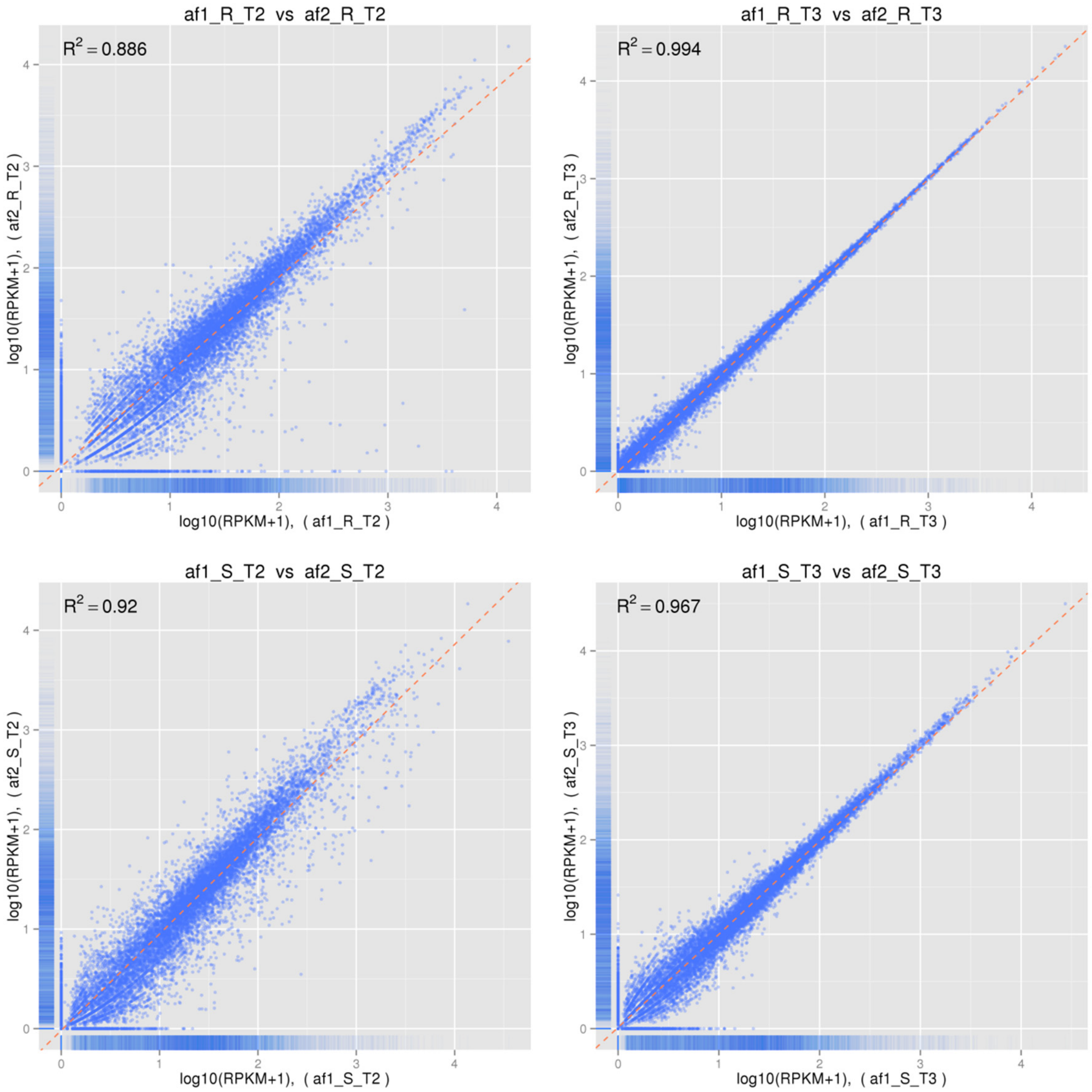

Figure S3. Results of the pearson correlation analysis of biological replicates.

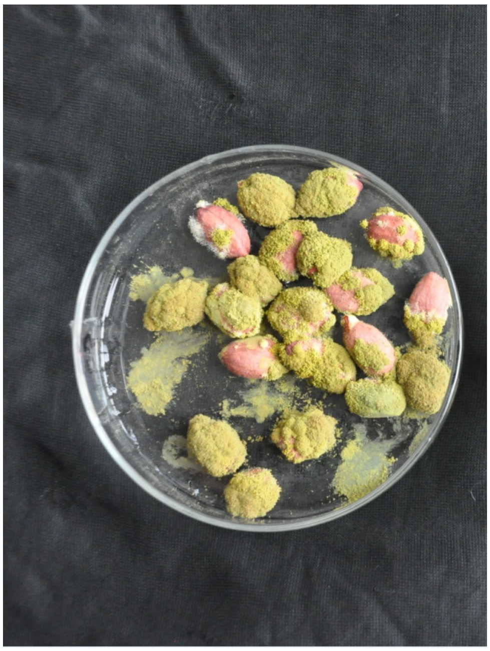

***A. flavus*-Zhonghua 12**

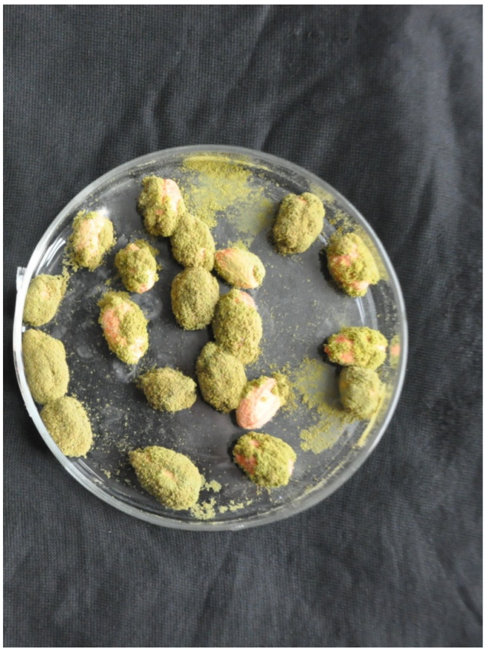

***A. flavus*-Zhonghua 6**

Figure S4. The morphological observation of *A. flavus*-peanut pathosystems at 7 d after incubation.

Table S4. Distribution of gene expression levels in *A. flavus*.

Acount	Library Name	RPKM					
		0–1	1–3	3–15	15–60	>60	>1
Gene Number	af1_R_T2	3928	1030	3073	3703	2723	13,827
	af2_R_T2	4001	1335	2953	3610	2553	10,456
	af1_R_T3	3305	1510	3154	3857	2631	11,152
	af2_R_T3	3330	1505	3186	3828	2608	11,127
	af1_S_T2	3285	1354	3228	3905	2685	11,175
	af2_S_T2	3804	1391	3094	3663	2505	10,653
	af1_S_T3	3565	1482	3174	3668	2568	10,892
	af2_S_T3	3567	1473	3278	3596	2543	10,890
Proportion (%)	af1_R_T2	27.17	7.12	21.26	25.61	18.84	72.84
	af2_R_T2	27.68	9.23	20.43	24.97	17.69	72.33
	af1_R_T3	22.86	10.44	21.82	26.68	18.20	77.14
	af2_R_T3	23.03	10.41	22.04	26.48	18.04	76.96
	af1_S_T2	22.72	9.37	22.33	27.01	18.57	77.27
	af2_S_T2	26.31	9.62	21.40	25.34	17.33	73.69
	af1_S_T3	24.66	10.25	21.95	25.37	17.76	75.34
	af2_S_T3	24.67	10.19	22.67	24.88	17.59	75.33

Table S8. The aflatoxin content in *A. flavus*-Zhonghua 6 and *A. flavus*-Zhonghua 12 pathosystems at different days after incubation.

Incubation Time (d)	Aflatoxin Content (μg/kg)	
	A. flavus-Zhonghua 6	*A. flavus*-Zhonghua 12
1	0 ± 0	0 ± 0
3	3175.5 ± 232.8	12,687.1 ± 720.2
7	21,107.6 ± 1487.4	195,223.8 ± 14354.4
